# The impact of sex on blood pressure

**DOI:** 10.1097/MNH.0000000000001077

**Published:** 2025-04-07

**Authors:** Fanny Bourdon, Belen Ponte, Anne Dufey Teso

**Affiliations:** Nephrology and Hypertension Division, Geneva University Hospitals, Geneva, Switzerland

**Keywords:** cardiovascular risk, hormones, hypertension, sex differences, sex-specific treatment

## Abstract

**Purpose of review:**

Hypertension is the most prevalent cardiovascular disease worldwide and the leading cause of mortality in both men and women. Despite well documented sex differences in prevalence, risk factors, and treatment responses, current guidelines still fail to take these specificities into account. A more tailored approach, accounting for sex-specific pathophysiological mechanisms and risk factors, is essential.

**Recent findings:**

Studies show that hypertension is more prevalent in men than in women until menopause. After menopause, the prevalence increases in women, likely due to hormonal changes. Additionally, genetic, metabolic, and social risk factors differ between the sexes, as do cardiovascular risks and associated comorbidities. Pharmacokinetic and pharmacodynamic variations also impact antihypertensive treatment efficacy and side effects, highlighting the need for a more individualized therapeutic strategy. This review explores the pathophysiology of hypertension by sex, global risk factors with a focus on female-specific aspects, and sex-related cardiovascular risks. We also discuss antihypertensive treatments and their effectiveness based on gender-specific characteristics.

**Summary:**

Incorporating sex differences into hypertension management could enhance treatment efficacy and reduce cardiovascular mortality. Further research is needed to refine guidelines and develop personalized therapeutic strategies, optimizing hypertension care and improving patient outcomes.

## INTRODUCTION

Hypertension (HTN) affects approximately 1.28 billion adults worldwide, with significant sex differences in prevalence and trajectory across the lifespan [1]. While HTN is more common in men before the age of 50, women face an increased risk after menopause. The sex-specific ratio of HTN is estimated to affect approximately one in five women and one in four men in 2015. These differences are attributed to sexual dimorphism, both in terms of blood pressure (BP) control and regulation [2]. The sex difference is particularly pronounced in young adulthood between the ages of 18 and 29, where HTN is estimated to affect 1.5% of women compared with 5% of men [3]. This disparity is driven by biological, hormonal, and physiological factors.

Despite the long-standing recognition of sex differences in medicine, research on the pathophysiological distinctions between men and women remains limited. Sex is recognized as a key biological variable, which should encourage researchers to expand their investigations to better account for sex differences across a wide range of diseases, including HTN [2]. To improve HTN control and reduce the risk of cardiovascular disease, early identification of sex-specific risk factors and awareness is essential.

Therefore, in this review, we will describe the pathophysiology, risk factors, cardiovascular risk, and pharmacological differences between the sexes. 

**Box 1:**
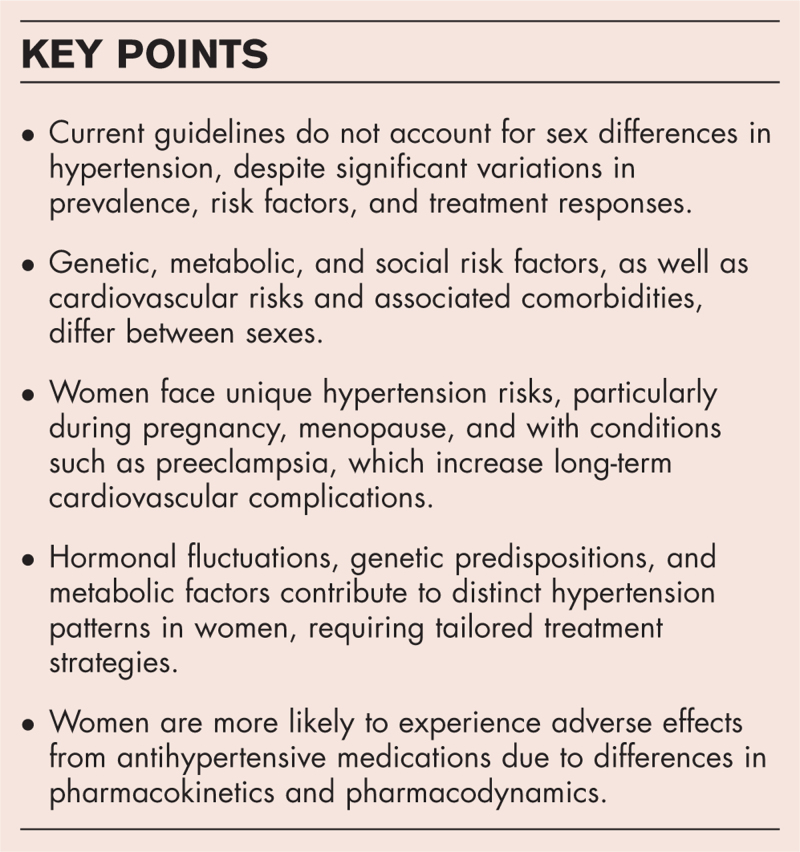
no caption available

## TRAJECTORY OF BLOOD PRESSURE OVER THE LIFE COURSE

The sex difference in the trajectory of HTN over the life course shows initially similar BP in early childhood. However, from the age of 7 years old onward, BP rises slightly faster in girls until the age of 12 and then slows down, with SBP being lower in young women than in young men [4]. At the age of 18, SBP is 10 mmHg higher in men compared to women. This difference remains stable until the age of 30. DBP follows a different curve after the age of 16, decreasing in both sexes, but more rapidly in men. Although men have higher SBP and DBP levels earlier in life, a crossover occurs in midlife. Beyond this period, women experience a more pronounced increase, particularly at menopause, with SBP rising by approximately 35%. At the age of 70, women BP reaches its peak, at which point the prevalence of HTN is higher than in men [5].

**FIGURE 1:**
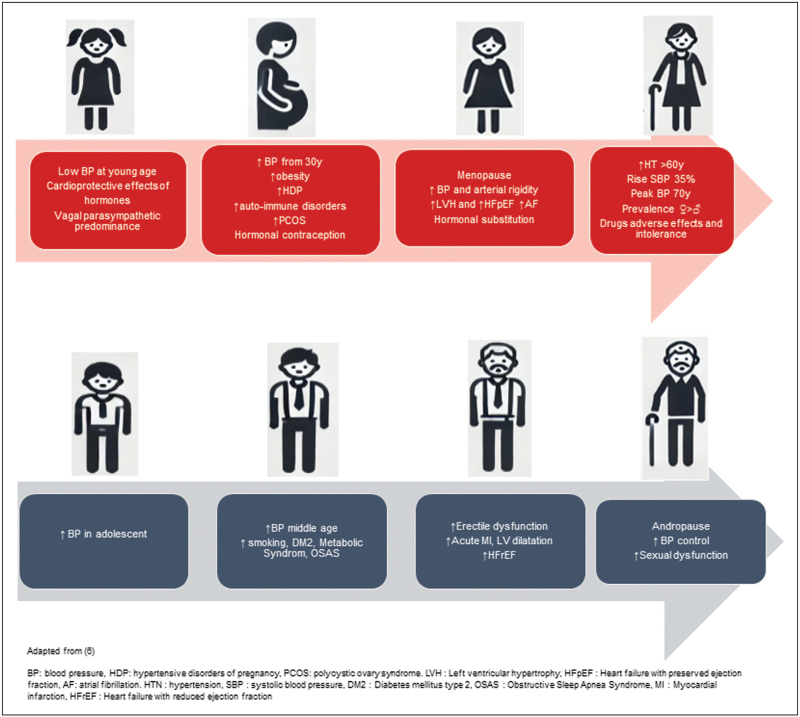
Sex differences in hypertension across life course.

Figure 1[6] shows how different HTN trajectories are across life course in men and women.

## PHYSIOPATHOLOGY OF HYPERTENSION IN MEN AND WOMEN

The regulators of BP and vascular function differ between women and men. These variations affect the autonomic nervous system, the renin–angiotensin–aldosterone system (RAAS), vasodilators such as bradykinin and nitric oxide, as well as hormonal and humoral mechanisms.

### Autonomic nervous system

Regulation of the autonomic nervous system varies between the sexes and throughout life [7^▪^]. Compared to healthy men, healthy women have a lower threshold for baroreceptor reflex sensitivity. In middle-aged, premenopausal hypertensive women, heart rate variability is altered, while vagal parasympathetic predominance is preserved. With aging and after menopause, the sympathetic system becomes dominant even in normotensive women, replacing the parasympathetic control that primarily regulates BP in younger women [8,9].

### Hormone's impact

Sex hormones play an important role in BP regulation. Estrogen and progesterone reduce the activity of the RAAS, while androgens stimulate it.

The RAAS may contribute to sex differences in HTN, as it exhibits higher activity in male individuals, potentially leading to adverse cardiovascular effects such as sympathetic activation, vasoconstriction, aldosterone release, and sodium retention [6].

In premenopausal women, estrogens provide cardioprotection by reducing calcium pathways in vascular, cardiac, and renal cells while regulating the genomic expression of vasoconstrictors, such as angiotensin II, endothelin-1, and catecholamines. Estrogens inhibit the RAAS by lowering plasma renin and angiotensin-converting enzyme activity. However, they also promote fluid retention by increasing angiotensinogen expression and elevating levels of angiotensin and aldosterone. Progesterone is a potent aldosterone antagonist and counterbalances this effect on sodium retention [6].

This explains the marked increase in sensitivity to sodium chloride and the enhanced synthesis of vasoconstrictors following menopause. Additionally, the upregulation of angiotensin II AT1 receptor expression exacerbates oxidative stress, thereby influencing vascular resistance and renal hemodynamics in postmenopausal women.

### Other factors

Genetic factors are also important, particularly the presence or absence of the Y sex chromosome. Indeed, its genetic variants are thought to modulate sympathetic nervous system activity, as well as sodium and potassium excretion, thereby influencing BP regulation. Additionally, some genes on the X chromosome are thought to contribute to oxidative stress [10].

Furthermore, women generally have smaller hearts and blood vessels, even when adjusted for body size. This anatomical difference can influence blood flow and impact HTN development [11]. Vascular stiffness can be measured by the velocity of arterial pulse waves traveling through the arterial tree, namely pulse wave velocity. The measurement of carotid–femoral pulse wave velocity confirms an increase in arterial stiffness with age in both sexes. However, women exhibit a more rapid increase in stiffness after the onset of menopause, consistent with estrogen reduction [8].

## RISK FACTORS OF HYPERTENSION

HTN is influenced not only by biological and behavioral factors but also by social determinants. Common risk factors for both men and women include age, family history, ethnic background, overweight/obesity, sedentary lifestyle, sodium chloride consumption, alcohol intake, smoking, and stress. Additionally, social factors such as low health literacy, limited access to healthcare, and inadequate preventive measures also play a role [12].

Risk factors for HTN are multiple and impact men and women differently, as illustrated in Table 1.

**Table 1:** Hypertension risk factors according to sex

		Female	
HTN risk factors	Male	Premenopause	After menopause
Age	+	(+)	++
Hormones	+	−	++
Vascular rigidity	+	−	++
Salt sensitivity	+	+ (+)	+++
Metabolic syndrome	++	+	++
Visceral obesity	++	+	+ (+)
Type 2 diabetes	+(+)	+	+(+)
Dyslipidemia	++	+	++
Gout	+++	+	++(+)
Smoking	++	+	+
Autoimmune disorders	+	+++	+(+)

Adapted from [6]. HTN, hypertension; -, protective; (+), variable effect; +, common; ++, more common, +++, much more common.

Cardiometabolic factors such as obesity, insulin resistance, glucose intolerance, diabetes, dyslipidemia, hepatic steatosis, and sleep apnea are often exacerbated by a sedentary lifestyle and an unbalanced diet. All these factors are increasing in both sexes, with a clear male predominance. However, typical menopausal changes lead to an increase in visceral fat mass and alterations in lipid and procoagulant profiles, including elevated lipoprotein (a) levels, which heighten metabolic risk in postmenopausal women [13].

Salt sensitivity of BP (SSBP) is defined as a 10% variation in BP in response to salt consumption. It is a pathophysiological and hereditary trait that would account for approximately 50–80% of essential HTN diagnoses. Although SSBP is common in men and women, many large population studies have demonstrated that SSBP prevalence is higher in women [14].

Ethnic background may also influence the effects of sex hormones on BP, as the determinants of salt sensitivity appear to differ between Black and White hypertensive women. Black hypertensive women tend to experience a greater mean BP increase in response to salt loading compared to their White counterparts.

Moreover, as seen in a prospective multicenter study [15], smoking remains more prevalent in men, as does hyperuricemia and the risk of gout. Conversely, inflammatory and autoimmune diseases, such as systemic lupus erythematosus, are more prevalent risk factors in women.

## WOMEN-SPECIFIC HYPERTENSION RISK FACTORS

**FIGURE 2:**
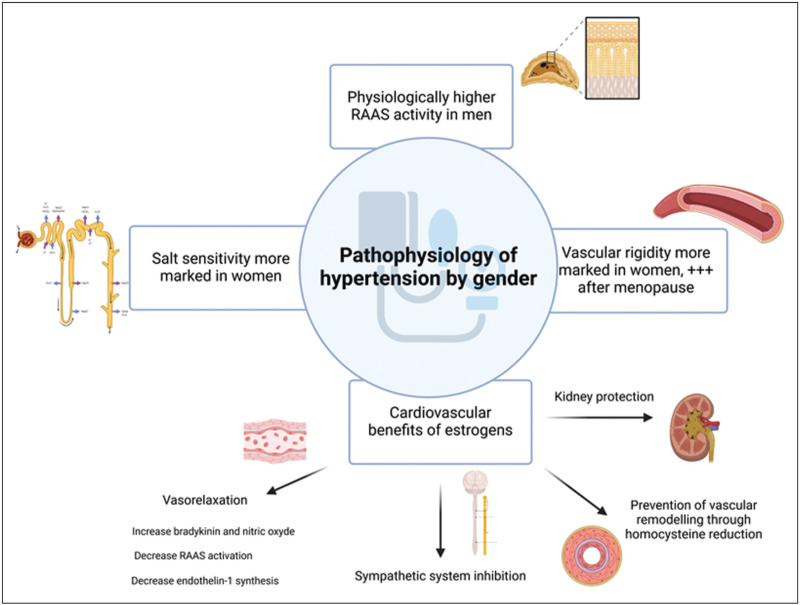
Pathophysiology of hypertension considering women specificities.

In addition to the conventional risk factors for HTN, several sex-related cardiovascular risk factors have been identified and need to be considered. Indeed, women undergo numerous hormonal changes throughout their lives, which can impact on their hypertensive and cardiovascular risk [16^▪▪^]. Figure 2[17] summarizes some of the pathophysiology of sex-related differences in HTN, focusing on women aspects.

### Menarche

Early or late menarche can be associated with HTN, as can menstrual disorders (premenstrual syndrome, painful or irregular periods).

### Gynecological disorders

Gynecological disorders, such as like polycystic ovary syndrome, fibroids, endometriosis, contribute to an increased cardiovascular risk and a higher likelihood of developing HTN well before menopause.

### Oral contraceptives

Oral contraceptives containing ethinylestradiol are associated with an increase in BP and cardiovascular and thromboembolic risk. It is, therefore, recommended to opt for low-dose contraceptives, transdermals or progestins alone, especially in cases of uncontrolled HTN [18].

### Pregnancy-related disorders

Pregnancy acts like a cardiovascular stress test, potentially triggering or revealing HTN. Hypertensive disorders of pregnancy include gestational HTN, preeclampsia, HELLP syndrome (hemolysis, elevated liver enzymes, low platelet count), or intrauterine growth retardation with low birth weight. These disorders not only elevate fetomaternal morbidity during pregnancy but also have long-term health consequences [19]. The cardiovascular risk is thus increased not only for the mother but also for the child later in life. The importance of obstetrical history in women's life course should be part of the systematic anamnesis, as any hypertensive disorders during pregnancy are considered a risk factor for developing not only HTN in the following years but also cardiovascular disease [stroke, myocardial infarction (MI), and chronic kidney disease (CKD)].

### Obesity

Obesity is strongly associated with HTN in women. The neuroendocrine hormone leptin plays an important role in obesity by regulating food intake, metabolism, and fat distribution. Additionally, progesterone appears to amplify leptin-mediated endothelial dysfunction in obese premenopausal women through aldosterone and endothelial mineralocorticoid receptors. Leptin also seems to contribute to obesity in women with polycystic ovary syndrome or uterine fibroids [20].

### Menopause

As already described, menopause has a significant impact on cardiovascular risk, especially when it occurs early, due to the loss of sexual hormone protection.

Hormone replacement therapy can, therefore, modify BP depending on the type of estrogens administered: oral forms increase hepatic angiotensinogen, activating the RAAS and potentially raising BP, while transdermal estrogens appear to have a neutral or hypotensive effect [21].

## DIFFERENCE IN CARDIOVASCULAR RISK BY SEX

HTN is the primary risk factor for morbidity and mortality in women, responsible for one in five deaths in the United States [22]. HTN is the most impactful modifiable cardiovascular risk factor on mortality. However, the cardiovascular risk associated with HTN differs by sex. In men, it is the second leading cause of mortality after smoking [6]. The associated mortality is higher in women than in men, and better control of HTN could reduce cardiovascular mortality by 30.4% in men but by 38% in women [23].

In women, HTN is a more significant risk factor for acute coronary syndrome and heart failure with preserved ejection fraction (HFpEF) [16^▪▪^,^23^]. Women who have experienced gestational HTN or preeclampsia are also at increased risk [23]. According to the INTERHEART study, HTN is a more significant risk factor for MI in women than in men [24]. Another study involving patients aged 40–69 years without underlying cardiovascular disease, of whom 56% were women, demonstrated that although the incidence of MI is higher in men, the relative risk of infarction associated with HTN is 80% higher in women [25]. A Korean study, involving more than 6.4 million young adults, found a higher cardiovascular risk (ischemic stroke, MI) in hypertensive women [26]. Left ventricular hypertrophy, a prognostic marker of HTN, is twice as common and less modifiable by treatment in women [27]. HTN also leads to more pronounced vascular stiffness in women, exacerbated after menopause, and is less reducible by antihypertensive treatments than in men [6]. The macrovascular and microvascular consequences of HTN also differ by sex. Excessive stiffness and high pulsatility of blood flow are associated with microvascular damage, particularly in the brain and kidneys [6]. HTN is also a major risk factor for stroke, which is higher in women than in men [27].

Additionally, the cardiovascular risk associated with HTN manifests at lower BP thresholds in women. Indeed, an increase in SBP is linked to a heightened risk of cardiovascular disease at lower levels in women compared to men [28]. A 40 years old woman, with stage 1 HTN, doubles her risk of acute coronary syndrome, unlike men [29]. Despite a lower threshold risk, BP targets have not yet been adapted for women.

Regarding peripheral artery disease, women are often diagnosed later, with more diffuse and severe involvement of the femoropopliteal arteries [6]. Finally, HTN is a risk factor for chronic kidney disease, with a five-fold higher risk in men than in women [30].

In addition, nocturnal SBP is a stronger predictor of all-cause mortality and cardiovascular disease in women. Furthermore, the absence of nocturnal dipping is associated with an increased risk of cardiovascular disease only in women [6].

## TREATMENTS

### Unisex target of blood pressure control

Given the biological and pathophysiological differences between women and men, one might consider that the management of HTN should be adapted to improve the prevention and prognosis of cardiovascular diseases in women, as well as to tailor and personalize treatment.

Starting with HTN definition, one could question a universal definition, as BP is generally lower in women, but cardiovascular risk seems to appear at a lower BP threshold. Moreover, universal targets for BP could also be challenged, as there is no evidence that the same targets lead to the same cardiovascular risk reduction in men and women. Firstline treatment recommendations, or even the dosage of medications, have not been specifically tested by sex.

However, current guidelines do not recommend differential management of HTN in women, despite studies indicating significant variations in treatment responses. For example, the SPRINT study, which demonstrated a 27% reduction in all-cause mortality with intensive treatment, included only 30% women. Given the low representation of women and the short follow-up duration, questions arise regarding the applicability and generalization of lower treatment targets to both women and men [16^▪▪^].

### Influence lifestyle habits

Many modifiable risk factors, such as diet and physical activity, play a key role in the development of HTN. However, their impact also varies by sex, influencing the progression of the disease in different ways.

As discussed before, salt sensitivity is a major risk factor for HTN, particularly in postmenopausal women [16^▪▪^]. Studies have shown that women are more likely than men to develop severe HTN with excessive sodium intake [6,31]. On the other hand, salt sensitivity is associated with an increased risk of all-cause mortality only in men, suggesting distinct pathophysiological mechanisms [22]. Lifestyle plays a central role in managing HTN, with sex-differentiated responses. For example, the Mediterranean diet significantly reduces cardiovascular risk in women, while frequent consumption of fried foods multiplies the risk of HTN by 2.4. In contrast, whole grains and legumes specifically reduce the risk of HTN in women [32^▪▪^]. Diets low in sodium or high in potassium and dark chocolate have shown superior effectiveness in reducing HTN in women [32^▪▪^]. Furthermore, aerobic exercise has a more pronounced effect in women [33^▪^], while weight loss does not show significant differences between sexes [16^▪▪^]. Smoking cessation, adjustment of concomitant treatments (reduction of nonsteroidal anti-inflammatory drugs, choice of appropriate oral contraceptives) are also key strategies for better HTN control [16^▪▪^].

### Pharmacological differences

Pharmacokinetic and pharmacodynamic differences affect responses to antihypertensive treatments. In women, a higher gastric pH and slower gastric emptying alter drug absorption, while their higher fat-to-lean mass ratio impacts the distribution of both lipophilic and hydrophilic molecules [31]. Moreover, enzymatic activity varies: women show increased cytochrome P (CYP) 450 3A4 activity, responsible for the metabolism of many drugs, while CYP 2C19 activity is reduced by 60% under oral contraception [6].

Table 2 lists the main differences in pharmacokinetics and efficacy between the usual therapeutic classes.

**Table 2:** Main pharmacological differences in antihypertensive treatments

	Male	Female
Angiotensin-converting enzyme inhibitors	Similar efficacy Physiologically higher activity of the RAAS	Similar efficacy Lower activity of Enalapril at low concentrations More frequent cough and angioedema Contraindicated during pregnancy
Angiotensin II receptor blockers	Similar efficacy Physiologically higher activity of the RAAS	Similar efficacy Telmisartan's *C*_max_ higher in women, with no difference in BP Contraindicated during pregnancy
Thiazide diuretics	Higher risk of gout	Higher risk of arrhythmia, hyponatremia, and hypokalemia
Calcium channel blockers	Slower elimination	Greater effect of Amlodipine on BP in women compared to men Amlodipine shows a higher incidence of lower limb edema Appears superior to other therapeutic classes for stroke prevention, with a greater benefit in women Higher clearance of Verapamil due to greater CYP3A4 activity and lower P-gp activity
Beta-blockers	Erectile dysfunction	Premenopausal women are less sensitive to the vasoconstrictive effects of the adrenergic system via estrogen's influence on certain BB responses Reduced clearance of Metoprolol with more pronounced effects at similar doses Greater risk of side effects for beta-blockers metabolized by CYP 2D6 (Metoprolol, Carvedilol, Nebivolol, Propranolol)

BB, beta-blockers; BP, blood pressure; *C*_max_, peak concentration; CYP, cytochrome P450; P-gp, P-glycoprotein; RAAS, renin–angiotensin–aldosterone system.

### Inequalities in management and treatment adherence

Despite similar HTN prevalence in men and women, women are often less well treated or followed [18]. There are, however, differences in antihypertensive prescriptions: men are more often given angiotensin converting enzyme (ACE) inhibitors, angiotensin II receptor blockers (ARBs), and beta-blockers, while women are primarily treated with diuretics and calcium channel blockers, mainly due to pregnancy risks. [31]. Furthermore, women are underrepresented in clinical trials, limiting the knowledge of sex-based differences in treatment response [23]. Finally, psychosocial factors, such as depression and dissatisfaction with healthcare, are more common in older women and contribute to poorer management of their HTN. However, there is better treatment adherence in women than in men, with both genders showing improved adherence when the number of pills is reduced (polypill) [34].

## CONCLUSION

HTN is a major global health issue, with significant sex differences in prevalence, risk factors, and treatment responses. These should be taken into account when managing HTN.

Current guidelines do not sufficiently consider these differences, underscoring the need for a more personalized approach that accounts for sex-specific pathophysiological mechanisms and risk factors. A more personalized approach, considering female-specific risk factors, physiological differences, and treatment responses, could significantly improve outcomes for female patients with HTN.

The underrepresentation of women in scientific research, whether as professional researchers or as study participants, hinders the production and application of knowledge in clinical guidelines targeting women. Hopefully, the new scientific recommendations insisting on stratifying any analysis or study inclusion by sex will provide more evidence in the future and hopefully help in applying specific treatment trajectories for women in HTN.

Screening for HTN, regardless of gender, remains one of the key strategies for improving the early management of HTN. Further research is needed to refine guidelines and develop personalized therapeutic strategies that optimize HTN care and improve patient outcomes.

## Acknowledgements

*None.*

### Financial support and sponsorship

*None.*

### Conflicts of interest

*There are no conflicts of interest.*

## REFERENCES AND RECOMMENDED READING

Papers of particular interest, published within the annual period of review, have been highlighted as:

▪ of special interest
▪▪ of outstanding interest
